# Noise Source Visualization Using a Digital Voice Recorder and Low-Cost Sensors

**DOI:** 10.3390/s18041076

**Published:** 2018-04-03

**Authors:** Yong Thung Cho

**Affiliations:** Division of Mechanical and Automotive Engineering, Kongju National University, Cheonan, Chungnam 31080, Korea; cho.yong@gmail.com; Tel.: +82-41-521-9277

**Keywords:** low cost, digital voice recorder, sound visualization, noise source identification

## Abstract

Accurate sound visualization of noise sources is required for optimal noise control. Typically, noise measurement systems require microphones, an analog-digital converter, cables, a data acquisition system, etc., which may not be affordable for potential users. Also, many such systems are not highly portable and may not be convenient for travel. Handheld personal electronic devices such as smartphones and digital voice recorders with relatively lower costs and higher performance have become widely available recently. Even though such devices are highly portable, directly implementing them for noise measurement may lead to erroneous results since such equipment was originally designed for voice recording. In this study, external microphones were connected to a digital voice recorder to conduct measurements and the input received was processed for noise visualization. In this way, a low cost, compact sound visualization system was designed and introduced to visualize two actual noise sources for verification with different characteristics: an enclosed loud speaker and a small air compressor. Reasonable accuracy of noise visualization for these two sources was shown over a relatively wide frequency range. This very affordable and compact sound visualization system can be used for many actual noise visualization applications in addition to educational purposes.

## 1. Introduction

Accurate sound visualization of noise sources is required for optimal noise control. Measuring sound levels at only a few locations around a source region may not provide enough information for optimally reducing noise. Typically, noise measurement systems require microphones, pre-amps, an analog-digital converter, cables, a data acquisition system, etc., which may not be affordable for many potential users, especially if the measurement environment is not ideal, which may imply the possibility of loss or damage to instruments or sensors. Also, many such systems are not highly portable and may not be convenient to move or travel with. 

Handheld personal electronic devices such as smartphones and digital voice recorders with relatively lower costs and higher performance have become widely available recently. Even though such devices are highly portable, directly implementing them for noise measurement may lead to erroneous results since such equipment was originally designed for voice recording. The performance of smartphones has improved very rapidly, and they have practically become small computers since apps can be developed and executed on them. As a result, diverse efforts to implement smartphones as portable measurement devices have been made [1,2,3,4,5,6,7,8,9,10,11,12,13,14,15]. This includes application as low-cost noise measurement devices [16,17,18,19,20,21,22].

The performance of digital voice recorders has also significantly improved, especially in terms of sound quality, memory capacity, and battery life. The price range for digital voice recorders varies according to performance and functionality. However, relatively low-cost digital voice recorders with reasonable performance capabilities are readily available on the market. One of the advantages of using digital voice recorders for noise visualization is that they have separate microphone and headphone jacks, and the two channels of external microphones can be used conveniently. A minimum of two microphones are required for noise visualization: at least one microphone is required for reference measurement and one for scanning the sound field, as employed in this study. The external microphones used, which were low-cost electret condenser microphones, were connected to a digital voice recorder to perform measurements and the resulting content was processed for noise visualization. One of the challenges of using a digital voice recorder to measure sound pressure is that the microphone gain or amplification ratio may change as the sound amplitude changes. Another challenge of using a digital recorder with unit amplification for the microphone is that the recorded level of sound pressure may be so low that it is not possible to get audible sound pressure when replayed with the digital recorder if the source level is relatively low.

In this study, such characteristics of a digital recorder were investigated and sound visualization performance was investigated. A low-cost, compact sound visualization system was introduced and implemented to visualize two actual noise sources for verification with different characteristics: an enclosed loud speaker and a small air compressor. 

## 2. Sound Measurement Using a Digital Recorder

**Figure 1:**
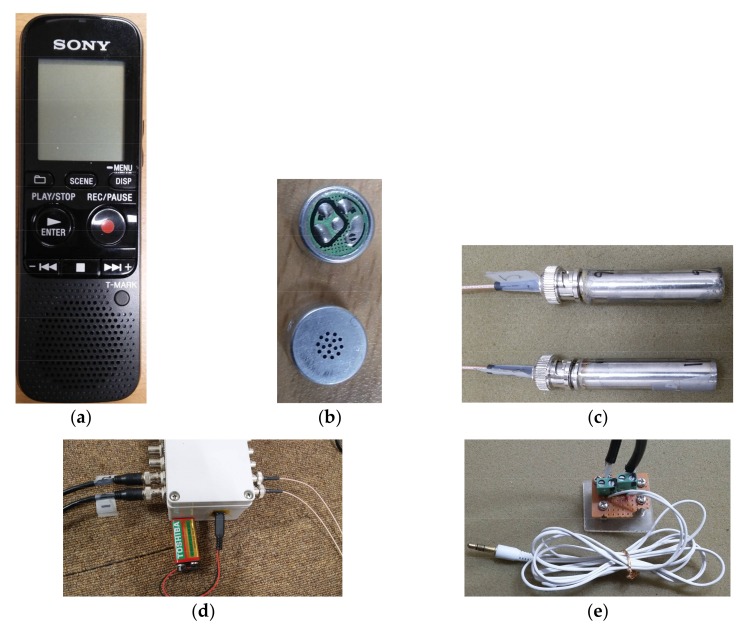
Digital recorder and microphones: (**a**) Digital recorder; (**b**) Electret condenser microphone capsules; (**c**) Electret condenser microphone capsules in tube (**d**) Electret condenser microphone power supply; (**e**) BNC to 3.5 mm plug adapter.

The price range of digital voice recorders is quite wide according to performance, design, functionality, etc. However, relatively low-cost digital voice recorders with reasonable performance are readily available on the market. In this study, a SONY IC Recorder ICD-PX312 (Sony Electronics Inc., San Diego, CA, USA), shown in Figure 1a, was used to record sound. However, the scope of this study was only to confirm that digital recorders can be used as low-cost sound visualization systems, and the performance of one digital recorder relative to others was not investigated in detail.

One of the advantages of using a digital voice recorder for noise visualization is the included 3.5 mm microphone jacks, which allow for an external microphone, the two channels of which can be used conveniently. More microphones are required for accurate measurement as the size and complexity of a source increases, especially for an incoherent sound field [23,24]. In the present study, two external microphones were used, one reference and one scanning. These were low-cost electret condenser microphones. The electret condenser microphone capsules used are shown in Figure 1b. These electret condenser microphone capsules were glued to one end of a 12.7 mm (half inch) outside diameter tube, and male BNC’s were glued to the other end, as shown in Figure 1c. These electret condenser microphone capsules and male BNC’s were connected with wires inside the tube by soldering.

One of the challenges of using a digital voice recorder to measure sound pressure is that the microphone gain or amplification ratio may change as sound amplitude changes. This characteristic of digital recorders is optimal for voice recording since this makes it possible to get more consistent and audible sound levels even with large fluctuations in sound input. However, this is not acceptable for a measurement device since it may cause different sound input levels to be recorded as similar sound pressure levels. To overcome this problem, the microphones were supplied with external power using a 9 V battery and connected to a digital recorder with unit amplification as shown in Figure 1d,e. Microphone tubes were connected to the microphone power supply input, and the output of the microphone power supply was connected with a 3.5 mm plug to the digital recorder via a 3.5 mm external microphone jack as shown in Figure 1d,e. By using the microphones’ external power supply, the digital recorder was provided with output from the microphones, similar to external devices for dubbing. In this study, the external microphones were not directly connected to the digital recorder without external power to prevent erroneous sound measurement due to adjustments of amplification gain during recording, which might happen during voice recording when not using an external microphone power supply. The digital recorder, microphones, power supply, and cables shown in Figure 1 were used for all measurements in this study unless specified otherwise. 

**Figure 2:**
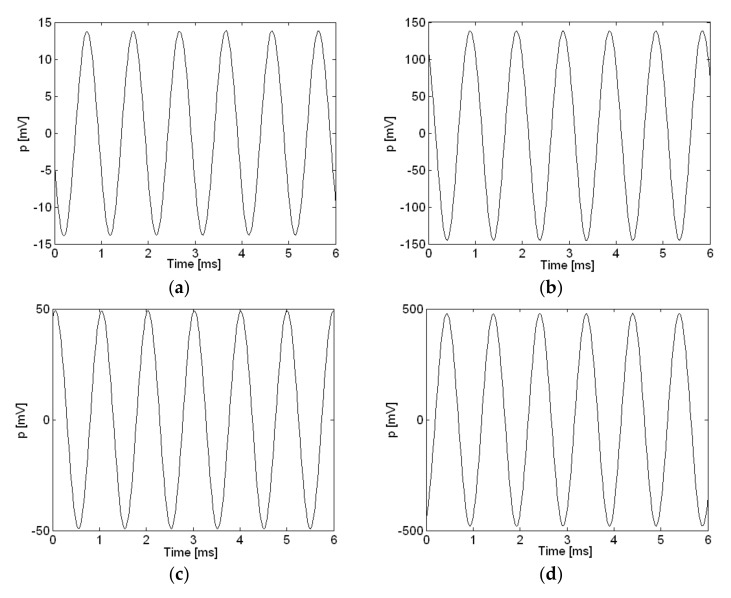
Calibration of microphones at 94 dB and 114 dB at 1000 Hz: (**a**) Microphone channel 1, 94 dB; (**b**) Microphone channel 1, 114 dB; (**c**) PCB microphone, 94 dB; (**d**) PCB microphone, 114 dB.

For measurement, sound was recorded at the highest quality available with the digital recorder. The frequency range for this highest setting was between 75 Hz and 20 kHz [25]. Also, the noise cut filtering of the digital recorder was turned off during measurement to prevent any unwanted filtering of data. Both microphone channels were calibrated at 1 kHz and 94 dB sequentially using external sound calibrator, CEM model SC-05 (CEM, Shenzhen Everbest Machinery Industry Co., Ltd., Nunshen, Shenzhen, China), and the digital recorder saved the data as mp3 files. These files were then transferred to a desktop computer using a USB cable, converted from mp3 to wav file format, and saved as data files in MATLAB (MathWorks, Natick, MA, USA) to be processed as measurement data. The measurement sampling rate was 44.1 kHz. The measurement for the calibration of microphone channel 1 at 1 kHz, 94 dB and 114 dB is shown as uncalibrated measurement data with units in mV in Figure 2a,b. Linearity of the microphone response was confirmed from 1 kHz up to 114 dB as shown in Figure 2a,b. Also, the calibration measurement results of a PCB microphone, model 378B02 (PCB Piezotronics, Inc., Depew, NY, USA), are shown for comparison with the results from the electret condenser microphones. The measurement setup for the PCB microphone was similar to that of the electret condenser microphones, as shown in Figure 1, except the actual microphone and signal conditioner model PCB 482C (PCB Piezotronics, Inc., Depew, NY, USA). Measurements for the calibration of the PCB microphone channel at 1 kHz, 94 dB and 114 dB are shown in Figure 2c,d. Linearity of the PCB microphone response was confirmed from 1 kHz up to 114 dB as shown in Figure 2c,d. The sensitivity of the PCB microphone was more than three times greater than that of the electret condenser microphones used in this study. The PCB microphone was much more expensive but also a better tool for measurement than the electret condenser microphones. Nonetheless, it was confirmed that the electret condenser microphones employed here represent a low-cost, usable option, and further investigation was deemed to be required to confirm measurement accuracy for sound visualization. By combining a digital recorder with electret condenser microphones and an external power supply, the lowest possible cost was incurred and a conveniently portable sound visualization system was constructed.

## 3. Visualization of Sound Measurement

An acoustical holography procedure [26], which also could be described as an inverse system procedure, was implemented to visualize noise sources such as an enclosed loudspeaker and a small air compressor. Acoustical holography was first introduced for projection of measurements with spherical coordinates [27]. A version using planar and cylindrical coordinates was later introduced to allow measurement surfaces to conform more closely with source geometry [28,29]. An alternative holography procedure, statistically optimized for near-field acoustical holography (SONAH), was later derived to reduce spatial truncation and the size of the measurement surface [30,31]. SONAH was also modified for the projection of measurements with cylindrical coordinates [32]. Cylindrical SONAH was implemented to visualize noise radiated from automotive power seat motors [33]. By using fixed reference signals during measurement, a scan can be completed with a relatively small number of microphones for successful acoustical holography reconstruction [23,24]. Three different types of measurement techniques such as cylindrical SONAH, a structural impulse caused using an impact hammer, and a motor run-up test were used to identify noise sources in a small DC motor [34]. In this study, planar SONAH [30,31] was implemented to visualize two different types of noise sources, a loudspeaker and a small air compressor, to confirm the accuracy of this low-cost sound visualization system.

Sound pressure on a plane (*x*, *y*, *z*) can be expressed as [31,35]:

(1) $$p(x,y,z)=\frac{1}{{\left(2\pi \right)}^{2}}\int_{-\infty }^{\infty }\int_{-\infty }^{\infty }P(K)\Phi_{K}\left(x,y,z\right)dK,$$

where *P*(**K**) is the wave number spectrum of *p*(*x*, *y*, *z*), and the wave number vector **K** is defined as **K** ≡ (*k_x_*, *k_y_*). The three-dimensional, planar wave function, *Φ*_K_(*x*, *y*, *z*), is defined as:

(2) $$\Phi_{K}(x,y,z)\equiv e^{i\left(k_{x}x+k_{y}y+k_{z}\left(z+d\right)\right)},\;z\geq z_{s}=-d$$

where *z_s_* defines the nominal source plane. Note also that the *z*-component of *k*, *k_z_* is,

(3) $$k_{z}=[\begin{array}{c} \sqrt{k^{2}-{\left|K\right|}^{2}}\;\mathrm{for}\;k\geq \left|K\right|\\ -i\sqrt{{\left|K\right|}^{2}-k^{2}}\;\mathrm{for}\;k<\left|K\right| \end{array}$$

with *k* = *ω*/*c*, *ω* being the angular frequency and *c* the ambient sound speed. Assume now that the complex sound pressure *p*(**r**_h,j_) has been measured at *J* positions, **r**_h,j_ ≡ (*x*_j_, *y*_j_, *z*_h_), on the hologram surface. The pressure *p*(**r**) at an arbitrary position **r** ≡ (*x*, *y*, *z*) in the source free region *z* > *z_s_*, is estimated as a linear combination of the measured sound pressure data, *p*(**r**_h,j_):

(4) $$p(r)\approx \sum_{j=1}^{J}c_{j}(r)\cdot p(r_{h,j}).$$

Thus in order to determine the coefficients *c_j_*, Equation (4) must provide a good estimate for a finite sub-set of the planar wave functions defined in Equation (2):

(5) $$\Phi_{Kq,m}(r)\approx \sum_{j=1}^{J}c_{j}(r)\Phi_{Kq,m}(r_{h,j}),\;\;m=1\;\ldots \;M,\;q=1\;\ldots \;N.$$

The various quantities involved in this calculation are defined in the form of matrices and vectors as:

(6) $$A\equiv \left[\Phi_{k_{zq},m}(r_{h,j})\right],\;\alpha (r)\equiv \left[\Phi_{k_{zq},m}(r)\right],\;c(r)\equiv \left[c_{j}(r)\right].$$

Equation (5) can then be expressed in matrix form as:

(7) α(r)≈A c(r).

The regularized least squares solution for the weight vector, **c**(**r**), is then:

(8) $$c(r)={\left(A^{+}A+\theta^{2}I\right)}^{-1}A^{+}\alpha (r),$$

where ^+^ denotes the Hermitian or conjugate transpose, **I** is the identity matrix, and the regularization parameter, *θ*, is:

(9) $$\theta^{2}={\left[A^{+}A\right]}_{ii}\;10^{-SNR/10},$$

where *SNR*, the regularization parameter, depends on the signal-to-noise ratio of the measurement signal and the reconstruction location from the measurement. The subscript *ii* is used here to denote the diagonal elements of a matrix. Reconstructed pressure, *p*(**r**), is expressed as:

(10) $$p(r)\approx \sum_{j=1}^{J}c_{j}(r)p(r_{h,j})=p^{T}(r_{h})c(r)=p^{T}(r_{h}){\left(A^{+}A+\theta^{2}I\right)}^{-1}A^{+}\alpha (r),$$

where **p**(**r***_h_*) is the vector of measured pressures.

The normal particle velocity on the reconstruction plane, *u_z_*(**r**), can be found using Euler’s equation:

(11) $$u_{z}(r)=\frac{1}{i\rho_{o}\omega }\frac{\partial p(r)}{\partial z}.$$

By substituting the estimated spatial distribution of the sound pressure from Equation (10) into Equation (11), the normal particle velocity, *u_z_*(**r**), can be obtained as:

(12) $$\begin{array}{ll} u_{z}(r) & \approx \frac{1}{i\rho_{o}\omega }\frac{\partial }{\partial z}\left[p^{T}(r_{h}){\left(A^{+}A+\theta^{2}I\right)}^{-1}\;A^{+}\alpha (r)\right]\\ & =p^{T}(r_{h}){\left(A^{+}A+\theta^{2}I\right)}^{-1}\;A^{+}\beta (r)=p^{T}(r_{h})d(r), \end{array}$$

where **d**(**r**) is the transfer matrix between measurement pressure and reconstructed particle velocity, and the vector ***β***(**r**) is defined as:

(13) $$\beta (r)\equiv \frac{1}{i\rho_{o}\omega }\frac{\partial \alpha (r)}{\partial z}=\left[\frac{1}{i\rho_{o}\omega }\frac{\partial }{\partial z}\Phi_{Kq,m}(r)\right].$$

The elements of ***β***(**r**) are modified planar wave functions, which by use of Equation (2) can be expressed as:

(14) $$\Phi_{Kq,m}^{u}(x,y,z)\equiv \frac{k_{z}}{\rho_{o}\omega }e^{i\left(k_{x}x+k_{y}y+k_{z}\left(z+d\right)\right)},$$

where *ρ_o_* is the ambient density. The vector ***β***(**r**) can then be written as:

(15) $$\beta (r)\equiv \left[\Phi_{Kq,m}^{u}(r)\right].$$

Now, pressure and particle velocity can be reconstructed on other planes, such as source planes, from the measurement pressure. More detailed and accurate information about a source can be obtained using reconstructed properties than with measurement pressure alone.

## 4. Measurement Description

**Figure 3:**
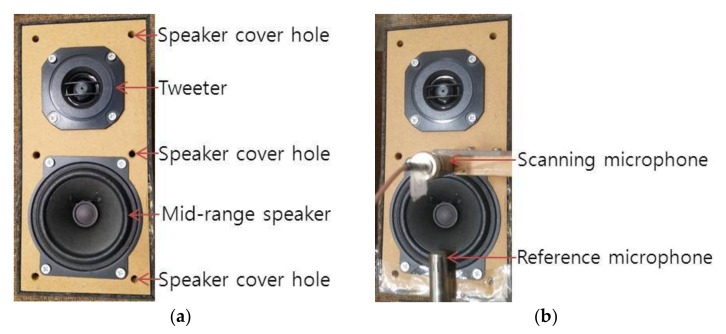
Loudspeaker and microphones used for noise measurement: (**a**) Loudspeaker; (**b**) Loudspeaker with reference and scanning microphones.

**Figure 4:**
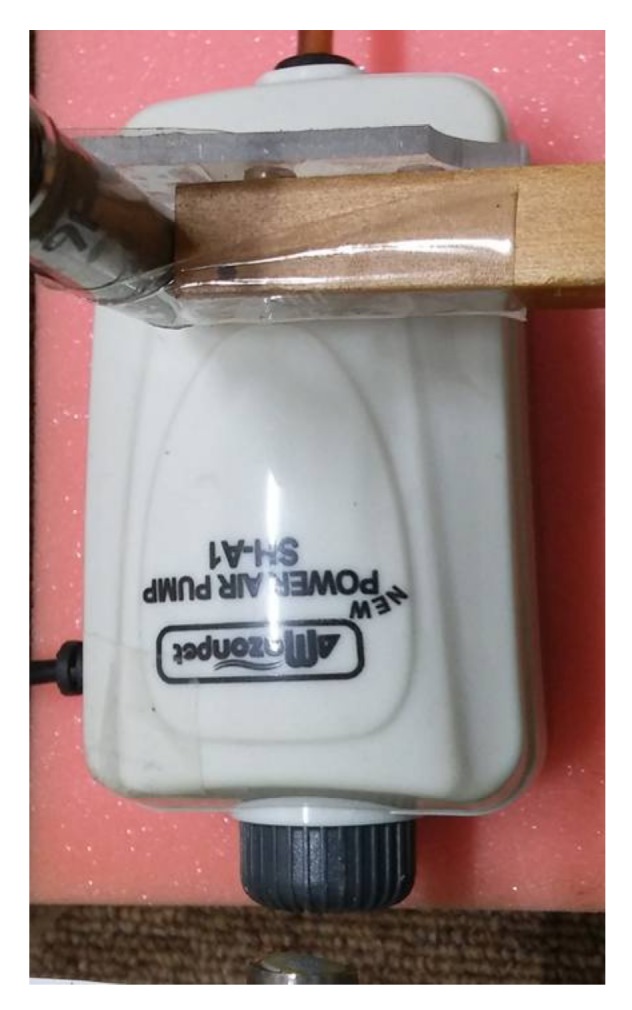
Small air compressor and microphones used for noise measurement.

**Figure 5:**
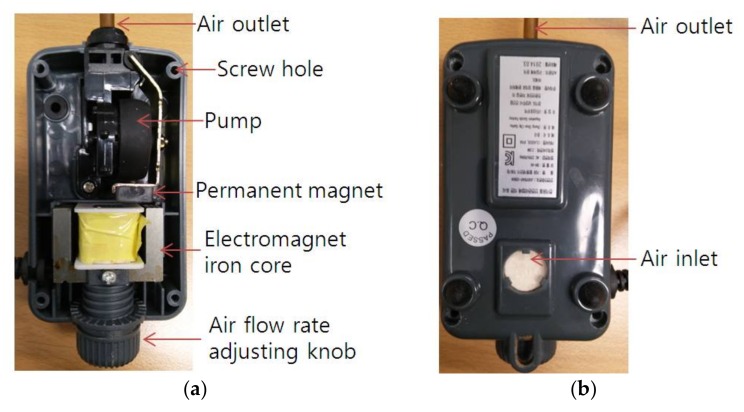

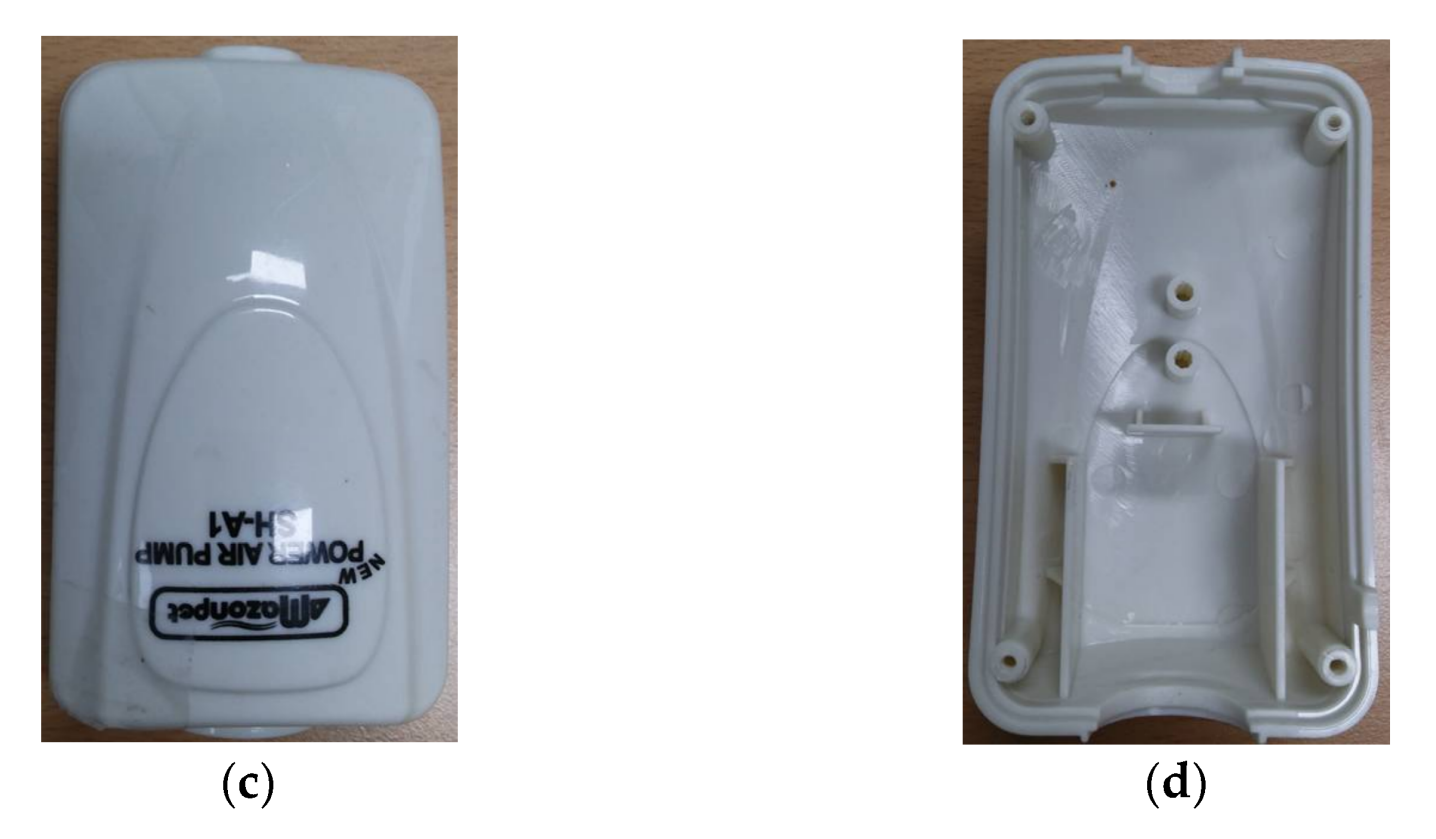
Inside and outside of air compressor and cover: (**a**) Inside of air compressor; (**b**) Bottom of air compressor; (**c**) Outside of air compressor cover; (**d**) Inside of air compressor cover.

The sound pressure of two sources with different characteristics was measured with two microphones, one reference and one scanning. First, the sound pressure of an enclosed loudspeaker driven by random noise was measured. The loudspeaker and microphones used for noise measurement are shown in Figure 3. The loudspeaker consisted of a mid-range speaker with a tweeter on top, as shown in Figure 3a. The exterior dimensions of the loudspeaker enclosure were 98, 200 and 95 mm, for width, height, and depth, respectively. Four surfaces of the loudspeaker enclosure, the top, bottom, and two sides, were made of 10 mm thick fiberboard. The stationary reference microphone is shown at the bottom of Figure 3b. Similarly, the sound pressure of a relatively quiet, small air compressor set to operate at a low flow rate was measured. The small air compressor and microphones used for measurement are shown in Figure 4. A reference microphone was located at the very bottom of the compressor, as shown in Figure 4. The compressor was situated on foam to prevent or minimize rattling noise from supports. The coordinate origins for both the loudspeaker and small air compressor measurements are located at the bottom left corners of Figure 3 and Figure 4, and the *x* and *y* axes coincide with the horizontal and vertical directions, respectively. Measurement increments are 2 cm for the loudspeaker and 1 cm for the small air compressor, with measurement conducted in both the *x* and *y* directions by locating scanning microphone manually over the measurement planes. The distance from the source and the measurement plane was 2 cm for the loudspeaker and 1 cm for the small air compressor. The surface of the air compressor curved concave down, so the distance from the measurement plane to the top of the air compressor surface was represented as the distance between the source surface and measurement plane. The loudspeaker produced six by eleven measurements, or 10 cm by 20 cm in the *x* and *y* directions. The small air compressor had a measurement size of seven by thirteen, or 6 cm by 12 cm in the *x* and *y* directions. Given the speed of sound in air being 343 m/s, to avoid spatial aliasing the highest frequencies considered were 8575 and 17,150 Hz for the loudspeaker and small air compressor measurements, respectively. The small air compressor sound pressure was measured under normal operation with air flow rate adjusted to the minimum level and the cover closed. The inside and outside of the air compressor and its cover are shown in Figure 5. The pump shown in Figure 5a was operated using electromagnetic force between a permanent magnet and an electromagnet iron core supplied with 220 V and 60 Hz, alternating current (AC) power. An air inlet was located in the bottom surface of the air compressor as shown in Figure 5b. An air outlet for the small air compressor was connected with a 0.9 m long open-ended plastic tube, of which the end was located relatively far from the air outlet.

**Figure 6:**
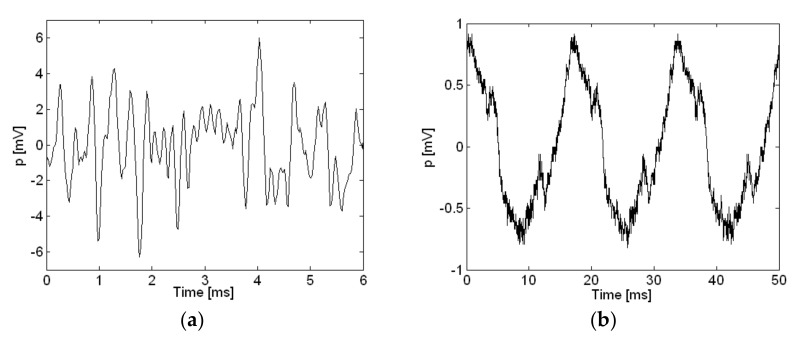
Reference sound pressure measurement in time domain: (**a**) Loudspeaker; (**b**) Air compressor.

An uncalibrated reference sound pressure measurement taken for the loudspeaker and small air compressor in the time domain with the same microphone is shown in Figure 6. The loudspeaker driven by random noise was a relatively louder sound source, and the small air compressor powered by 60 Hz AC was a relatively quiet sound source with different characteristics. In contrast to the loudspeaker, the measurement level for the small air compressor was very low when the reference measurements were replayed as mp3 files, turning out inaudible except for digital recorder noise. However, the mp3 files from the air compressor reference measurement were saved as data files and plotted as shown in Figure 6b, with periodic signals corresponding to about 60 Hz very clearly shown. This implies that even though the mp3 measurement files were not audible, the measurement data could be processed further for sound visualization and still yield reasonable results. Thus, the measurement data in the present work was processed further for sound visualization and shown. All signals in the present work were sampled at 44.1 kHz. The Hann window was applied to measurements from the microphones, and all measurement results shown in the frequency domain are A-weighted.

## 5. Measurement Results

**Figure 7:**
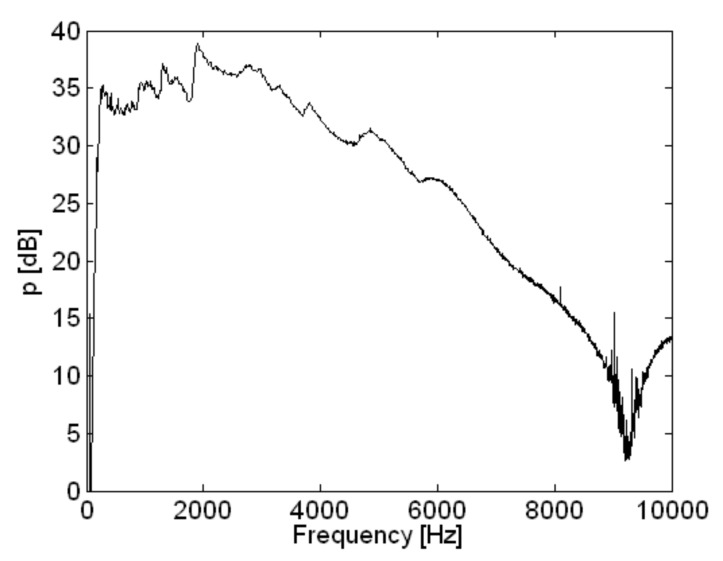
Spatially averaged measurement pressure of loudspeaker.

The spatially-averaged measurement pressure of the loudspeaker is shown in Figure 7. Deviation in the response of the loudspeaker was about 4 dB or less, and the response of the loudspeaker was relatively flat up to 4 kHz. The highest peak was at 1896 Hz as shown in Figure 7. However, the response of the loudspeaker decreased more rapidly above 4 kHz and had a minimum at 9204 Hz.

**Figure 8:**
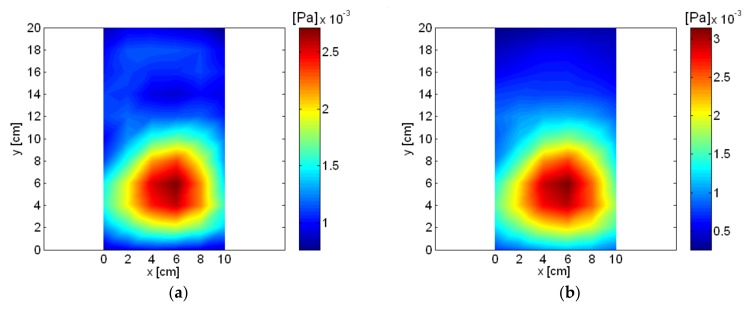

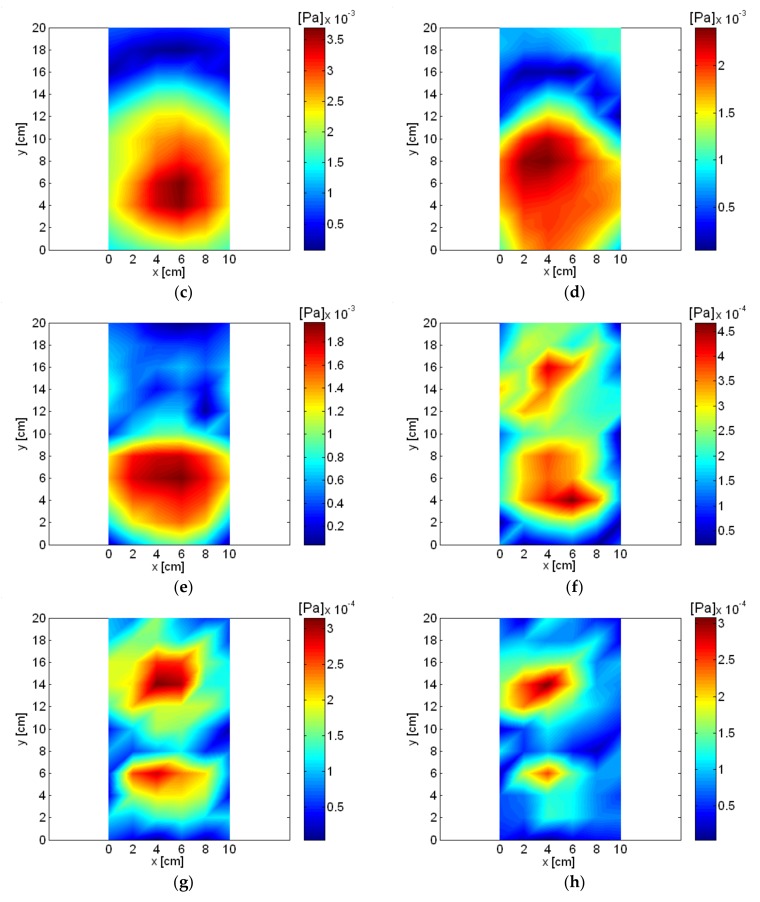
Reconstructed sound pressure of the loudspeaker: (**a**) 300 Hz; (**b**) 1000 Hz; (**c**) 1896 Hz; (**d**) 3000 Hz; (**e**) 4000 Hz; (**f**) 7000 Hz (**g**) 8000 Hz; (**h**) 8500 Hz.

**Figure 9:**
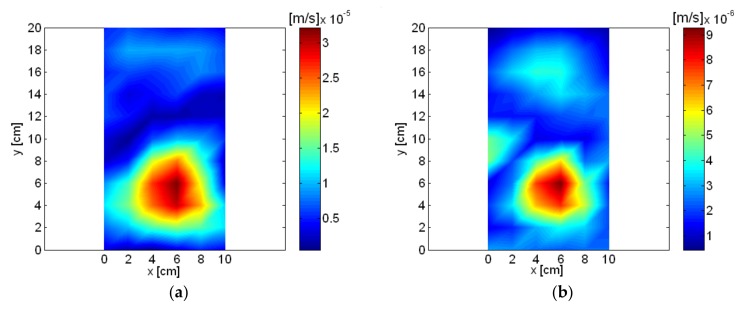

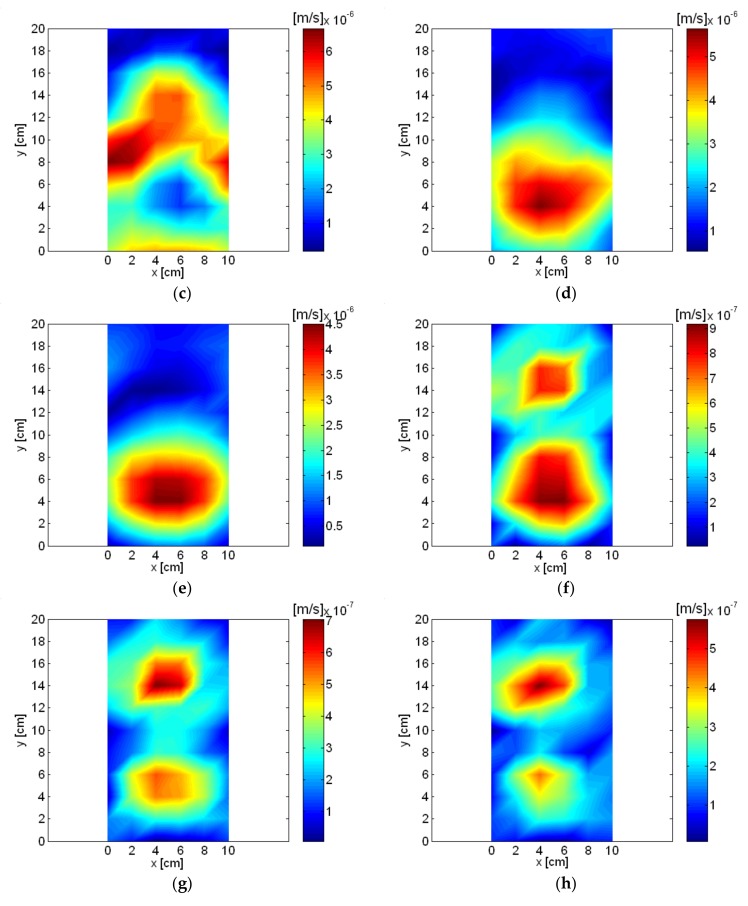
Reconstructed particle velocity of loudspeaker: (**a**) 300 Hz; (**b**) 1000 Hz; (**c**) 1896 Hz; (**d**) 3000 Hz; (**e**) 4000 Hz; (**f**) 7000 Hz; (**g**) 8000 Hz; (**h**) 8500 Hz.

Eight frequencies from the spatially-averaged measurement pressure of the loudspeaker were selected, and sound pressure and particle velocity were reconstructed for a source plane located 2 cm from the measurement plane. Among the frequencies of reconstructed sound pressure and particle velocity of the loudspeaker, the lowest frequency was 300 Hz. However, the highest frequency (avoiding spatial aliasing) was 8575 Hz for the loudspeaker measurement, and 8500 Hz was the highest frequency considered in reconstruction for the loudspeaker in this study. For selected frequencies, the reconstructed sound pressure and particle velocity of the loudspeaker on the surface plane are shown in Figure 8 and Figure 9. Except at 1896 Hz, the reconstructed sound pressure and particle velocity of the loudspeaker was similar up to a frequency of 4000 Hz, where the mid-range speaker was the dominant source. A significant difference between the reconstructed pressure and particle velocity appeared at 1896 Hz, which was the highest peak among the spatially-averaged measurement pressure data for the loudspeaker, as shown in Figure 7. Possibly due to internal acoustic resonance of the loudspeaker enclosure at 1896 Hz, a relatively complicated motion of sources was shown in the reconstructed particle velocity, which indicated a relatively large response from the front panel rather than the diaphragm of the loudspeaker. Considering the exterior dimensions of the loudspeaker enclosure, especially the height of 200 mm, and with the largest interior dimension being 180 mm, resonance at 1896 Hz was considered very reasonable. Sound radiation from the mid-range speaker was dominant at 3000 Hz and 4000 Hz, as shown in Figure 9d,e. Sound radiation from the tweeter was clearly shown at 7000 Hz or higher. The relative strength of the tweeter and mid-range speaker was more clearly shown in the reconstructed particle velocity than sound pressure. The transition of dominant sound sources is clearly visualized at frequencies above 7000 Hz in Figure 9f–h.

**Figure 10:**
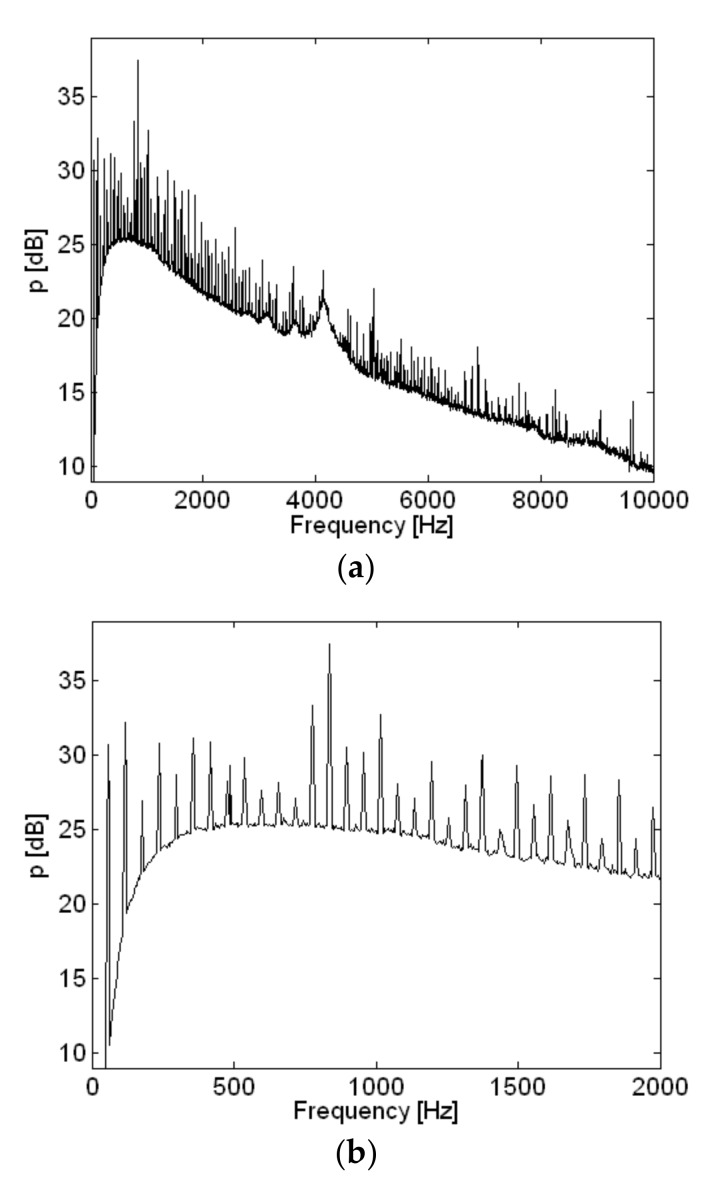
Spatially averaged measurement pressure of air compressor with different frequency ranges: (**a**) 0 to 10,000 Hz; (**b**) 0 to 2000 Hz.

**Figure 11:**
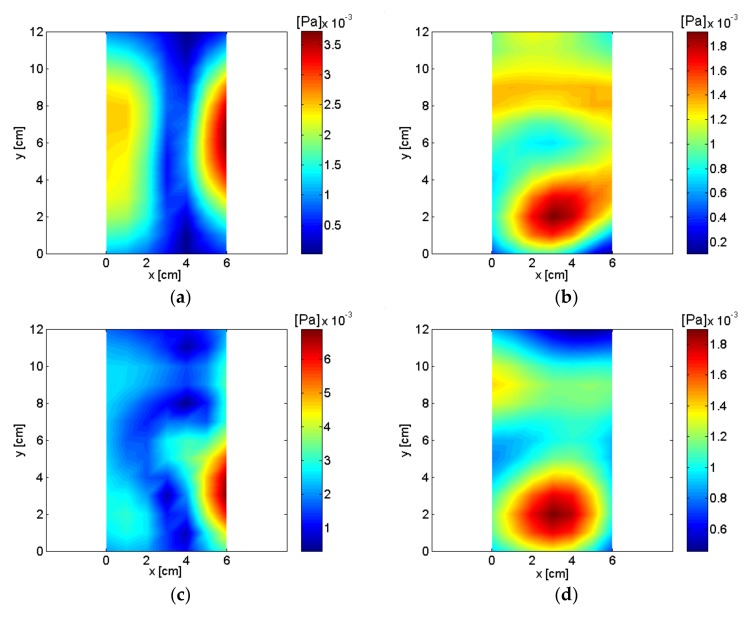

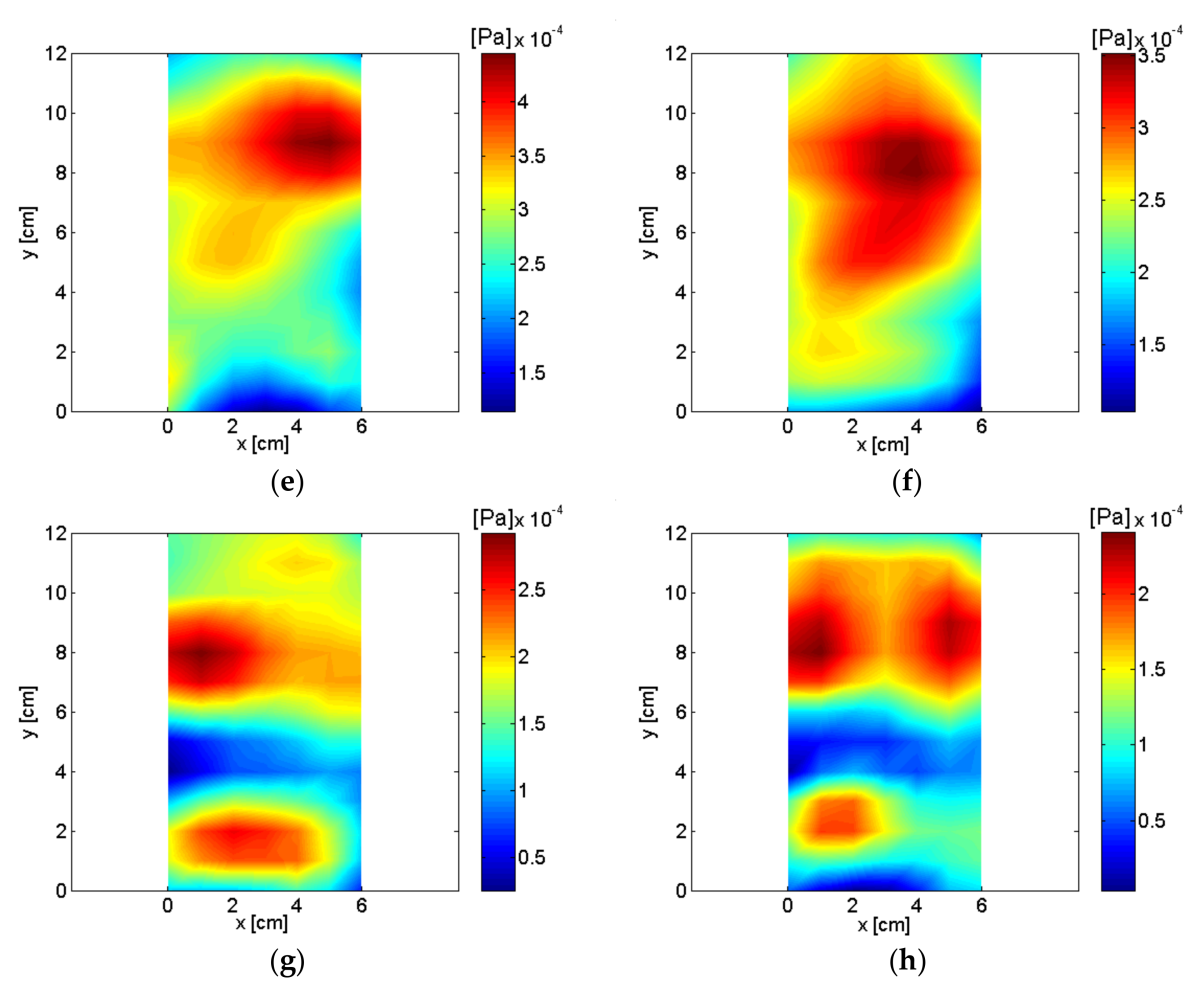
Reconstructed sound pressure of air compressor: (**a**) 56 Hz; (**b**) 356 Hz; (**c**) 836 Hz; (**d**) 1016 Hz; (**e**) 4132 Hz; (**f**) 5036 Hz; (**g**) 6888 Hz; (**h**) 8264 Hz.

**Figure 12:**
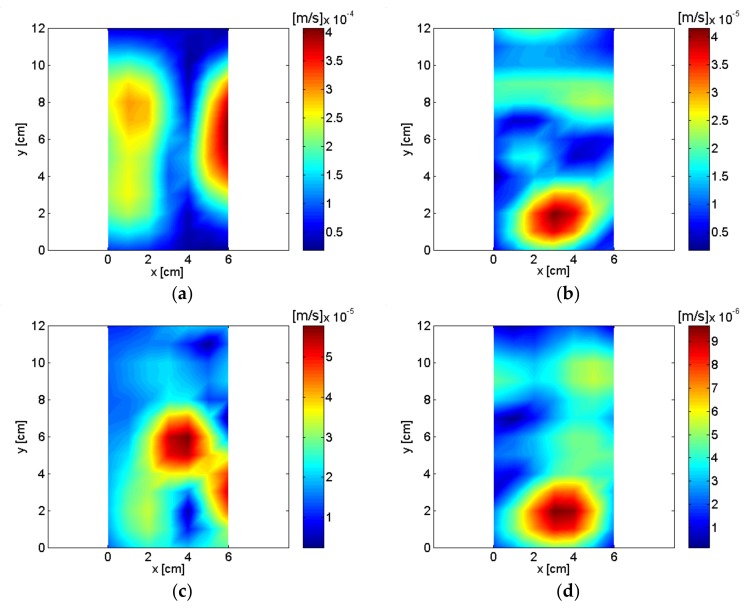

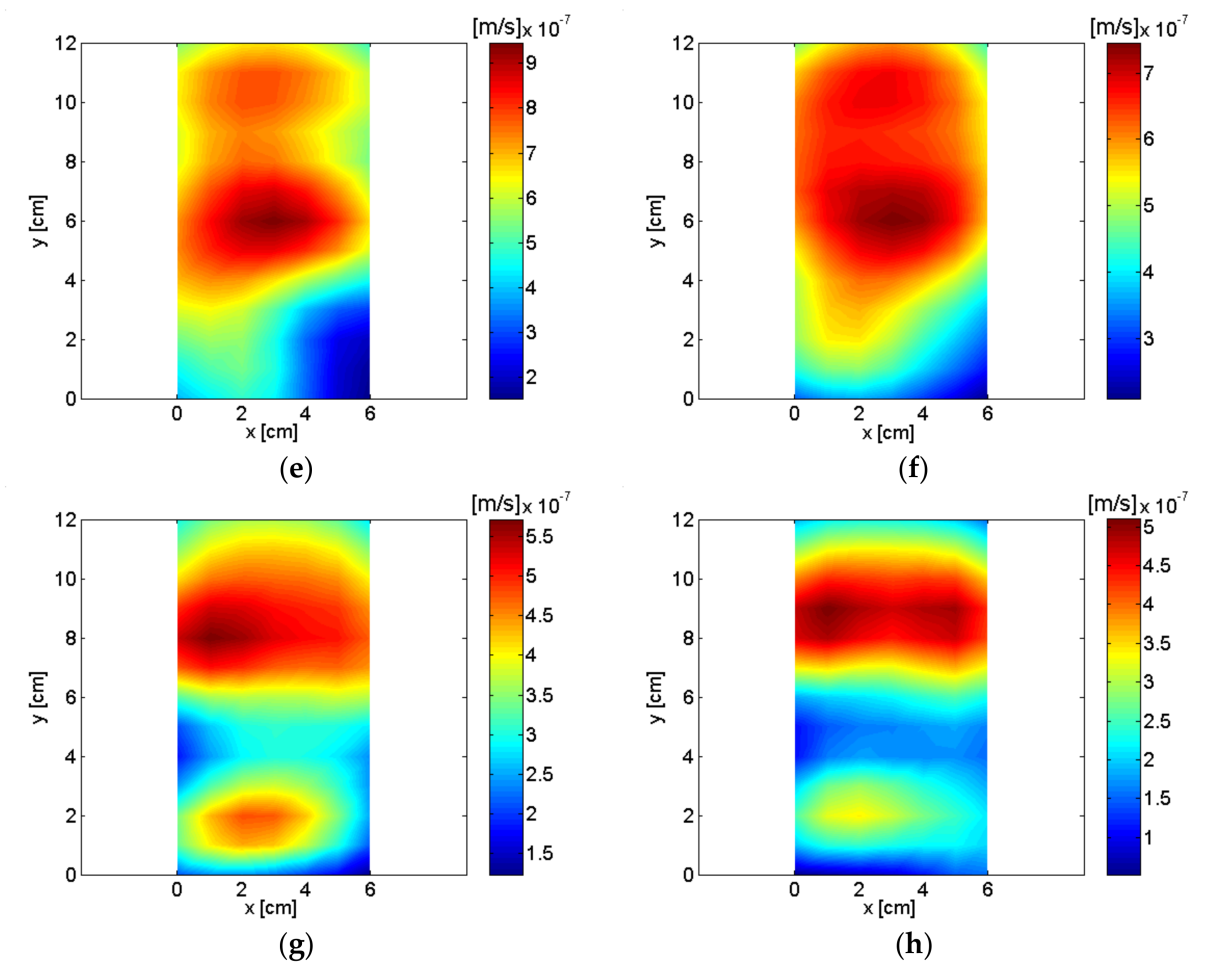
Reconstructed particle velocity of air compressor: (**a**) 56 Hz; (**b**) 356 Hz; (**c**) 836 Hz; (**d**) 1016 Hz; (**e**) 4132 Hz; (**f**) 5036 Hz; (**g**) 6888 Hz; (**h**) 8264 Hz.

The spatially-averaged measurement pressure of the small air compressor is shown in Figure 10. The same results with a different frequency range are shown in Figure 10a,b. Peaks appeared in the spatially-averaged measurement pressure at multiples of about 60 Hz, which is shown very clearly in Figure 10b. The highest spatially-averaged measurement pressure peak appeared at 836 Hz. Eight frequencies, including the highest peak, were selected, and the reconstructed sound pressure and particle velocity on a plane very close to the source are shown in Figure 11 and Figure 12. Rotational vibration of the entire body of the small air compressor, probably due to the oscillation of the permanent magnet at fundamental frequency, appeared at 56 Hz. Even though the air inlet was located in the bottom surface of the air compressor, it was a major source at 356 Hz and clearly emerged in the reconstructed particle velocity. At 836 Hz, which was the highest peak in the spatially-averaged measurement pressure of the small air compressor, oscillation of permanent magnet was a major source and was clearly visualized in the reconstructed particle velocity. Oscillation of the electromagnet iron core by reaction force was also a source at 836 Hz. The air inlet was a major source at 1016 Hz.

Oscillation of the permanent magnet and the air outlet were major sources at 4132 Hz and 5036 Hz. The pump and air inlet were major sources at 6888 Hz and 8264 Hz. Sources for the small air compressor are described and summarized in Table 1 based on reconstructed pressure and particle velocity on plane close to the source. Even though the cover of the small compressor was closed and it was adjusted to operate at a low air flow rate for quiet operating conditions, sources for the air compressor were visualized clearly from the measurements of the digital recorder.

## 6. Conclusions

In this study, a highly portable, low-cost sound visualization system was introduced by implementing a digital voice recorder and electret condenser microphones. External microphones, low-cost electret condenser microphones, were connected to a digital voice recorder to perform measurements and the recorded data was processed for noise visualization. One of the challenges of using a digital voice recorder for the measurement of sound pressure is that the microphone gain or amplification ratio may change as the sound amplitude changes. To overcome this problem, microphones were supplied with external power and connected to a digital recorder with unit amplification. Another challenge of using a digital recorder with unit amplification was that the recorded sound pressure was so low it was not possible to get audible results when the source level was relatively low. Nonetheless, a remarkably low-cost, compact sound visualization system was introduced through this study and implemented to visualize two actual noise sources for verification with different characteristics: an enclosed loudspeaker and a small air compressor. The sound measurement level for the small air compressor was relatively low and not audible when replayed with the digital recorder. However, reasonable accuracy of noise visualization for the two different noise sources was shown over a relatively wide frequency range.

The lowest and highest frequencies of reconstructed sound pressure and particle velocity for the loudspeaker were 300 and 8500 Hz, which represents almost full range of frequencies for the mid-range speaker and tweeter in this study. The transition of dominant sound sources from the mid-range speaker to the tweeter was clearly visualized for frequencies above 7000 Hz. The relative strength of the tweeter and mid-range speaker was more clearly shown in the reconstructed particle velocity than sound pressure.

The highest peak in the spatially-averaged measurement pressure of the small air compressor occurred at 836 Hz due to oscillation of the permanent magnet, which was very clearly visualized. Even though the cover of the small compressor was closed and it was adjusted to operate at a low air flow rate for quiet operating conditions, the sources for the air compressor were visualized clearly from the digital recorder measurements.

One of the advantages of using a digital voice recorder for noise visualization was that it had 3.5 mm microphone jacks, and the two channels of an external microphone could be used conveniently. The scope of the present study was to confirm that digital recorders can be used as low-cost sound visualization systems. Accuracy when combined with externally-powered, low-cost electret condenser microphones was confirmed qualitatively by comparison of experiment results from the characteristics of sources over wide frequency range. This very affordable, compact sound visualization system can be used for many actual noise visualization applications in addition to educational purposes.

## Figures and Tables

**Table 1:** Description of sources for the small air compressor.

Frequency	Order	Description of Source
56 Hz	1st	Rotational vibration
356 Hz	6th	Air inlet
836 Hz	14th	Permanent magnet oscillator (PMO)
1016 Hz	17th	Air inlet
4132 Hz	69th	PMO and air outlet
5036 Hz	84th	PMO and air outlet
6888 Hz	115th	Pump and air inlet
8264 Hz	138th	Pump and air inlet
